# Evaluation of health-related quality of life and its domains in pediatric patients with cancer

**DOI:** 10.1186/s43046-023-00168-1

**Published:** 2023-04-17

**Authors:** Mai Sabry Saleh, Asmaa Mahmoud Mohammed, Dina Bassiouni, Hend Helmy Mostafa, Zeinab Mohammed Monir

**Affiliations:** 1 Department of Environmental and Occupational Medicine, National Research Centre, Giza, Egypt; 2 Department of Pediatric Oncology, National Cancer Institute, Cairo University, Giza, Egypt; 3 Department of Child Health, National Research Centre, Giza, Egypt

**Keywords:** Health-related quality of life, Pediatric oncology, PedsQL™ Cancer Module

## Abstract

**Background:**

Health-related quality of life has emerged as a significant component in pediatric oncology research during the last several decades. Measures of health-related quality of life provide a thorough assessment of the child’s response to medical therapy, disease course, and adjustment outcomes in the context of pediatric oncology.

**Methods:**

The aim of the present study was to assess the cancer-specific health-related quality of life in cancer pediatric patients and to evaluate the contribution of its domains and some of the anthropometric, sociodemographic, and treatment-related variables on the overall quality of life, by using the PedsQL™ 3.0 Cancer Module.

**Results:**

The study included 110 cases. The mean value of the PedsQL™ 3.0 Cancer Module score was 49.3 ± 12.0. The lowest mean score of quality of life was for the “procedure anxiety” (8.7 ± 23.9), followed by the “worry” domains (16.6 ± 28.5). Higher “frequency of hospital visits” was associated with increased feeling of pain and treatment anxiety yet decrease in suffering from nausea and vice versa. The longer period of hospital admission for more than half of the recommended treatment period was associated with reduced pain suffering on the expense of increase in feeling of worry as well as communication problems. The perceived physical appearance was better among those patients who spent a treatment period for 3–6 months when compared to those who spent a treatment period less than 3 months or more than 6 months. There was a highly significant association between all the eight-cancer-specific quality-of-life domains except the pain domain- and the overall quality-of-life log scores. Nausea problem followed by worry and cognitive problems was the most effective domains on the overall quality-of-life score.

**Conclusion:**

Cancer pediatric patients suffered low quality of life especially for anxiety procedure and worry domains with special consideration for the impact of nausea, worry, and cognitive problems on their perception of quality of life.

## Background

Childhood cancer is a priority in Egypt due to the large number of children with cancer, particularly in the current resource-limited settings [1]. Egypt is a middle-income country, where 40% of the population are children under 18 years old (38.9 million in 2017) [2]. Age-standardized incidence rates of cancer in Egypt are 166.6 per 100,000 persons, and the 5-year survival of childhood cancer was estimated to be 40% as reported in 2008, based on baseline assessment of pediatric oncology care in Egypt [3]. The 5-year survival for children with cancer reported from Childhood Cancer Hospital of Egypt (CCHE) between 2007 and 2017 was 72.1% [4].

The available evidence shows that almost all cancer patients, including those with blood cancers, experience a complex array of challenges that threaten their physical, psychological, and spiritual wellbeing [5]. Cancer not only affects patients physically but also it may show a negative impact on the quality of life (QoL) for both cancer patients and survivors [6].

Health-related quality of life (HRQoL) is defined as the status of physical, emotional, and psychological wellbeing [7]. A direct association has been reported between higher HRQoL among patients under treatment and their regular school attendance and consequently their learning and educational attainment [8]. Moreover, identifying the health-related factors that affect the quality of life in cancer pediatric patients could allow the identification of patients who may benefit from early interventions and/or referral to psychological supportive programs [9]. In general, QoL assessment enables comprehensive evaluation of an illness interference with an individual adaptive functioning with consideration of person’s values, perspectives, satisfaction, living conditions, accomplishments, functionality, cultural context, and spirituality [8].

Most previous researches on QoL have focused on survivorship period of the pediatric cancer patients and much less addressed the period during active treatment phase. Moreover, there is an area of lacking knowledge about the influential effect of the health-related factors on the overall quality of life in age-specific cancer pediatric patients. Consequently, the objective of this work was to assess the cancer-specific HRQoL and to evaluate the contribution of its domains and some of the anthropometric, sociodemographic, and treatment-related variables on the overall quality of life.

## Methods

*The aim of the study* was to assess the cancer-specific HRQoL and to evaluate the contribution of its domains and some of the anthropometric, sociodemographic, and treatment-related variables on the overall quality of life.

### Patients’ recruitment

This analytical cross-sectional study has been conducted in the Outpatient Oncology Clinic of the National Cancer Institute, Egypt (NCI). A consecutive sample of the outpatient attendants for treatment/follow-up of different types of cancer in four consecutive months with age 8–12 years old have been included in the study. Exclusion criteria include cancer patients with critical conditions (disease relapse, severe neutropenia, treatment toxicities, psychological disturbances, etc.), congenital abnormalities, or intellectual disabilities. A total of 110 parents have participated to this study after completing a consent form explaining the aim of this work and declaring their right to withdraw from the study whenever they want. Institutional research ethics approval was obtained from the National Research Centre Committee to perform the study.

### Methodology

All patients were subjected to the following:

- ▪ *Full history taking*: Clinical sheet was completed, and full history was taken from the parents in addition to data extracted from medical records, laying stress up on.
  - *Demographic data* (as name, age, sex, residence)
  - *Medical history of cancer*: Including diagnosis, date of diagnosis, age at diagnosis, frequency of hospital visits, hospitalization period, and duration of treatment
  - *Past history* of other diseases or operations
  - *Family history* of cancer or other diseases or congenital abnormalities
  - *Child’s previous growth and development*, with precise timing and sequence of the physical milestones and cognitive functions
  - Vaccination history was included.
- ▪ *Clinical examination* of all body systems has been done.
- ▪ *Anthropometric indices* have been evaluated, using standardized equipment, and following the recommendations of the International Biological Program [10]
  - Height was measured by Harpenden stadiometer in centimeters, weight measured by Tanita scale, and body mass index (BMI) calculated according to the equation as follows: $BMI=\mathrm{weight}\;\mathrm{in}\;\mathrm{kg}/{\mathrm{height}\;\mathrm{in}\;\mathrm{meters})}^2$  
  - BMI percentile for age and sex (2–20 years old) was calculated electronically using Omni calculator software [11].

### Instrument

Quality of life was assessed using the parent proxy form of the Pediatric Quality of Life Inventory (PedsQL™) version 3.0 Cancer Module for the age categories 8 to 12 (Arabic version) [12]. It is composed of 27 items comprising 8 dimensions: pain and hurt, nausea, procedural anxiety, treatment anxiety, worry, cognitive problems, perceived physical appearance, and communication problems. Each of these dimensions was evaluated using 5-point Likert scale from 0 (never) to 4 (almost always), and then, items were reversed scored and linearly transformed to a scale range from 0 which indicates poor health-related quality of life to 100 which indicates good quality of life, i.e., higher scores of the problem on the scale indicate lower problems.

### Statistical analysis

The collected data have been analyzed using IBM SPSS version 20.0 software. Shapiro–Wilk test for normality was used to detect the significant deviation from the normal distribution. The continuous variables were presented as mean ± SD, median, interquartile range (IQR), and total range. For skewed data, Mann–Whitney *U*-test was used for comparisons between two independent groups and Kruskal–Wallis *H*-test for more than two groups; the results were expressed as mean ranks. The higher mean rank indicates the lesser health problem. The correlation between two variables was detected using bivariate Pearson’s correlation coefficient (r) and point**-**biserial coefficient** (***r*_**pb**_**)**. After log transformation of the data, multiple linear regression modeling was performed to detect the associations of different health-related quality-of-life domains, as well as each individual item, with the overall quality-of-life score. These associations were presented as *β*-coefficients ± standard error and 95% confidence interval after adjusting the potential confounding factors. All tests of significance were two sided, and statistical significance was defined as *P* < 0.05.

## Results

### Patients’ characteristics

The age range of patients in this study was 8–12 years old with mean age 10.0 ± 1.4 and median age 10.0 years old. As shown in Table 1, children < 10 years old represent 38.2% (*n* = 42), and children ≥ 10 years old represent 61.8% (*n* = 68). Male patients represent 60.9% (*n* = 67) of the sample, and an overall of 60.9% were living in rural areas. The majority of patients (60.34%) had normal weight (5th–85th), while 10.34% were underweight (< 5th), 13.79% were overweight (85th–95th), and 15.52% were obese (≥ 95th). The mean value of the BMI percentile is shown to be 55.4 with 34.1 as standard deviation and with interquartile range (IQR) from 27.7 to 88.9.

**Table 1:** Differences in cancer-specific quality-of-life domain scores by demographic and clinical characteristics (*n* = 110)

Character	Pain & hurt	Nausea	Procedure anxiety	Treatment anxiety	Worry	Cognitive problems	Communication problems	Perceived physical appearance	TSQL
	MR/P	MR/P	MR/P	MR/P	MR/P	MR/P	MR/P	MR/P	MR/P
**Age group (years)**									
**< 10 y (*n* = 42)**	48.6/0.05	53.0/0.5	51.5/0.09	54.7/0.8	53.8/0.6	49.7/0.1	56.5/0.7	56.2/0.8	50.9/0.2
** ≥ 10 y (*n* = 68)**	59.7	56.9	57.9	56.0	56.5	59.0	54.8	55.0	58.3
**Gender**									
**Male (*n* = 67)**	53.3/0.3	56.6/0.6	54.0/0.3	53.4/0.4	55.2/0.9	54.2/0.5	51.8/0.1	58.5/0.2	53.2/0.3
**Female (*n* = 43)**	58.8	53.7	57.7	58.6	55.9	57.5	61.2	50.8	59.0
**BMI status (percentile)**									
**Underweight (< 5th)**	34.5/0.2	31.5/0.5	28.5/0.5	31.4/0.1	26.0/0.6	26.1/0.7	27.1/0.1	26.5/0.3	29.5/0.08
**Normal (5th–85th)**	30.2	28.3	29.3	32.6	29.4	30.8	31.1	32.2	32.8
**Overweight (85th–95th)**	30.8	36.3	28.5	26.3	29.3	26.2	36.2	29.3	29.3
**Obese (≥ 95th)**	22.1	26.7	31.7	18.6	32.1	29.2	18.7	21.1	16.5
**Residence**									
**Urban (*n* = 43)**	52.6/0.8	55.4/0.6	54.2/0.7	54.5/0.7	54.6/0.7	57.6/0.2	52.2/0.7	53.4/0.9	53.8/0.9
**Rural (*n* = 67)**	54.0	52.3	53.0	52.9	52.8	51.1	54.4	53.5	53.3
**Age at diagnosis**									
** < 6 y (*n* = 27)**	49.5/0.5	55.8/0.5	51.6/0.8	50.8/0.7	49.5/0.5	58.2/0.3	58.6/0.2	43.8/0.1	53.3/0.8
** ≥ 6 y (*n* = 83)**	53.2	51.6	52.7	52.9	53.2	51.0	50.9	54.7	52.2
**Freq. of visits**									
** < 2 visit/m (*n* = 69)**	59.8/0.02*	50.7/0.04	55.6/0.6	60.6/0.01*	53.9/0.5	55.0/0.9	55.9/0.6	53.0/0.4	57.4/0.2
** ≥ 2 visits/m (*n* = 41)**	46.6	62.4	53.9	45.2	56	54.8	53.3	58.3	50.7
**Adm. period**									
** < Half ttt period (*n* = 21)**	34.4/0.001*	64.0/0.1	58.7/0.2	58.6/0.4	68.4/0.008*	57.4/0.5	68.8/0.01*	54.5/0.9	57.2/0.6
** ≥ Half ttt period (*n* = 89)**	57.9	51.9	53.0	53.0	51.0	53.3	51.0	53.8	53.3
**Duration of treatment**									
** < 3 m (*n* = 15)**	37.4/0.053	55.6/0.8	59.3/0.5	50.8/0.6	62.7/0.4	61.0/0.05	54.8/0.7	54.8/0.04*	54.7/0.9
**3–6 m (*n* = 18)**	56.5	57.8	54.2	49.2	54.9	38.9	51.5	71.3	55.7
** ≥ 6 months (*n* = 77)**	57.1	53.5	53.7	56.3	52.9	56.7	50.7	50.7	54.1
**Cancer type**									
**ALL (*n* = 63)**	58.3/0.2	54.8/0.7	55.2/0.8	56.7/0.6	56.0/0.8	60.1/0.06	58.9/0.1	55.7/0.9	58.3/0.2
**Others (*n* = 47)**	51.7	56.4	55.8	53.9	54.7	49.3	50.8	55.1	51.6

*TSQL* total score for quality of life, *BMI* body mass index, *ALL* acute lymphocytic leukemia, *P* *P*-value, *significant (*P* < 0.05), **highly significant (*P* < 0.01)

The mean age of patients at cancer diagnosis was 7.5 ± 2.5 years old, and the majority of them (75.4%) were diagnosed at school age (≥ 6 years old). The majority of cases were acute lymphocytic leukemia (ALL), 57.2% versus 42.8% of the other cancer types. The mean duration of treatment was 2.5 ± 1.9 years, and the majority (70.0%) have been under treatment for ≥ 6 months. Most of the patients (80.9%) were hospitalized for a period ≥ half of the decided treatment period (*n* = 89). The “frequency of hospital visits” was ≥ 2 visits per month in 37.2% (*n* = 41) of the patients and < 2 visits per month in 62.8% (*n* = 69).

**Fig. 1:**
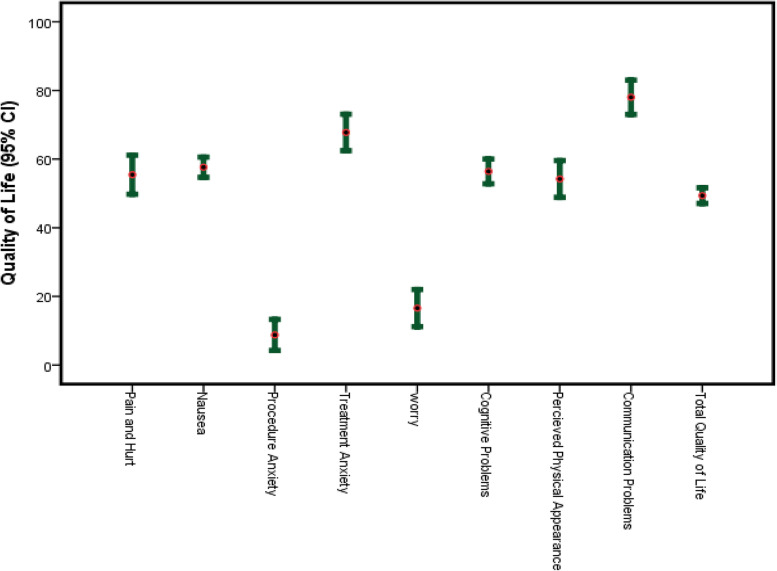
95% confidence interval for the overall quality-of-life scores and its domains in patients

**Fig. 2:**
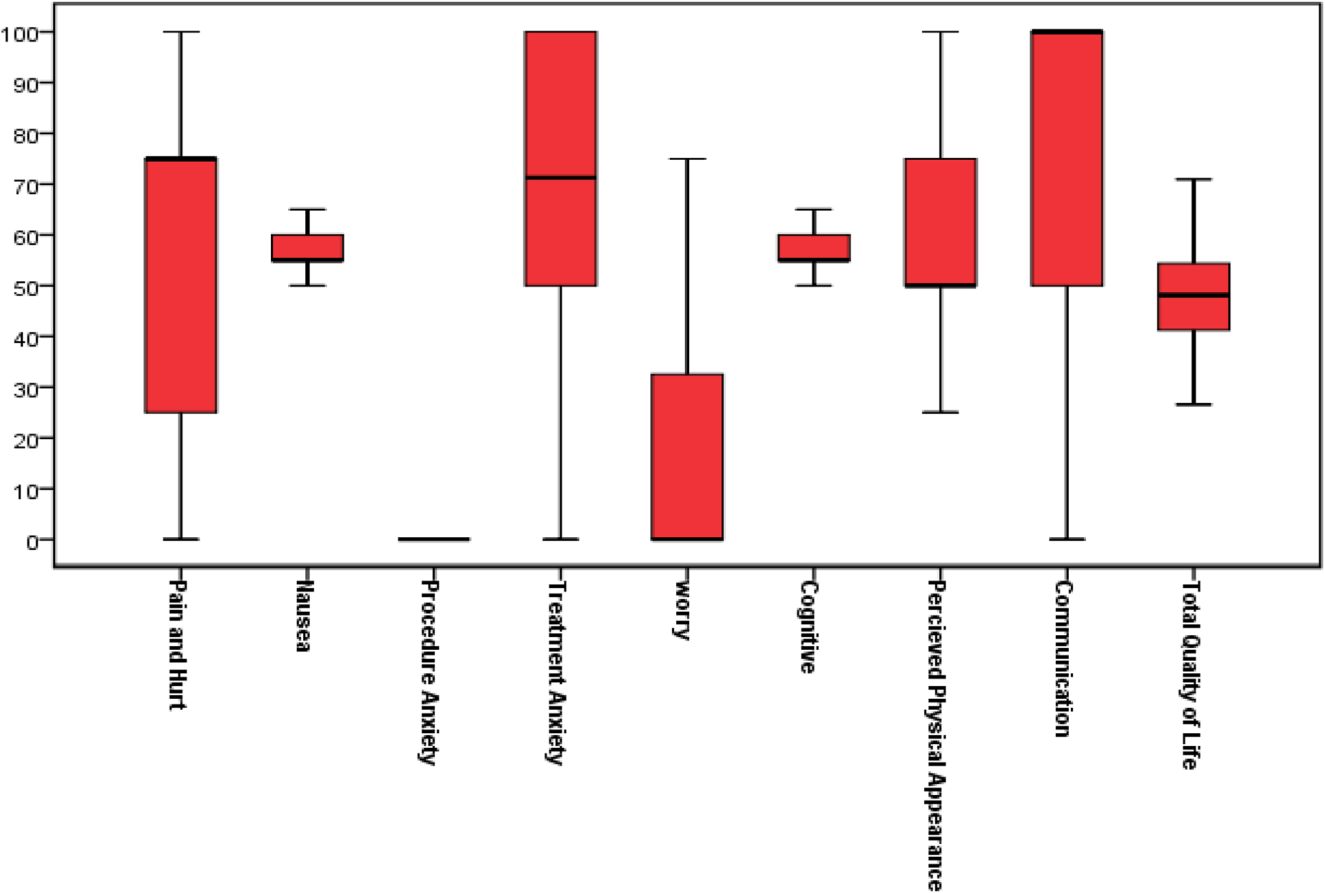
Distribution of the overall quality-of-life scores and its domains in pediatric cancer patients

As shown in Figs. 1 and 2, the mean score of the overall quality of life in pediatric cancer patients (TSQL) was 49.3, and median score was 48.1 with interquartile range (41.2–54.6) and total range (20.0–87.5) percent. The scores of the eight-cancer-specific quality-of-life domains were assessed; the total range of each domain ranged from 0.0 to 100.0.

The lowest mean score of quality-of-life domains was for the “procedure anxiety” followed by the “worry.” The mean score with standard deviation (M ± SD) for the “procedure anxiety” was 8.7 ± 23.9, and the median score was zero, while the mean value with standard deviation of the “worry” score was 16.6 ± 28.5, and the median score was zero. The M ± SD score of the “pain” was 55.4 ± 30.0, and the median score was 75.0 with IQR ranging from 25.0 to 75.0. The M ± SD score of “nausea” was 57.6 ± 15.5, and the median score was 55.0 with 55.0–60.0 IQR. The “treatment anxiety” showed M ± SD value of 67.7 ± 28.1 and median score of 71.2 with IQR of 50.0–100.0. The M ± SD of “cognitive problems” was 56.4 ± 19.1, median 55.0, and IQR of 55.0–60.0. The M ± SD score of “perceived physical appearance” was 54.2 ± 28.3, median score was 50.0, and IQR was from 50.0 to 75.0. Furthermore, the M ± SD score of “communication problems” was 78.0 ± 26.4, median score was 100.0, and IQR was from 50.0 to 100.0.

The statistical differences in the mean scores represented by the mean rank (MR) of the overall quality of life, as well as the eight QoL domains, were summarized in Table 1 based on the demographic and the clinical characteristics of the cancer pediatric patients. The patients, who visited the hospital < 2 visits/month for treatment/follow-up, showed significantly higher mean ranks of “pain” (59.8) and “treatment anxiety” (60.6) scores and lower mean rank (50.7) of “nausea” score. Thus, they complained less pain and treatment anxiety but felt more nausea than those who visited the hospital ≥ 2 visits/month (*P*-value = 0.02, 0.04, 0.01; respectively). In addition, the patients who were admitted to the hospital for a period ≥ half of the decided treatment period significantly suffered less pain (mean rank = 57.9) but more worry (mean rank = 51.0) and have more communication problems, in comparison with those admitted for a period < half of the decided treatment period (mean rank = 34.4, 68.4, 68.8; respectively); *P*-value (0.001, 0.008, 0.01, respectively). Moreover, there was significant perceived physical appearance problems among the patients with treatment periods ≥ 6 months (mean rank = 50.7) when compared to the treatment period 3–6 months (mean rank = 71.3; *P*-value = 0.04). Children with normal weight show higher mean ranks of total score of quality of life than obese or underweight children. No other significant differences among scores of quality-of-life domains, based on demographic and clinical characteristics, have been observed (Table 1).

The body mass index (BMI) and “frequency of patient visits” were significantly inversely correlated to the QL “treatment anxiety” domain score (*P*-value = 0.01 and 0.008, respectively). In addition, the “frequency of patient visits” also was significantly correlated inversely to the QL “pain” domain score, while it was correlated positively to the QL “nausea” domain score (*P*-value = 0.01 and 0.03, respectively). Both the “admission period” and the “duration of treatment” were significantly correlated positively to the QL “pain” domain score (*P*-value = 0.001, 0.01, respectively). These findings indicate that decrease in BMI of the patients as well as decreasing the number of patient visits as much as possible may improve the QL “treatment anxiety” domain score. No significant correlations were observed between the other patient characteristics and QL domains (Table 2).

**Table 2:** Correlations between cancer-specific quality-of-life domain scores by demographic and clinical characteristics of the studied children

Character	Pain and hurt	Nausea	Procedure anxiety	Treatment anxiety	Worry	Cognitive problems	Communication problems	Perceived physical appearance	TSQL
	*Coefficient (r)/p-value*	*Coefficient (r)/p-value*	*Coefficient (r)/p-value*	*Coefficient (r)/p-value*	*Coefficient (r)/p-value*	*Coefficient (r)/p-value*	*Coefficient (r)/p-value*	*Coefficient (r)/p-value*	*Coefficient (r)/p-value*
**Age (years)**	0.1/0.09	− 0.02/0.7	0.1/0.3	0.07/0.4	0.04/0.7	0.1/0.08	− 0.06/0.4	0.009/0.9	0.1/0.2
**BMI**	− 0.1/0.3	0.04/0.7	0.1/0.2	− 0.3/0.01*	0.01/0.9	0.04/0.7	− 0.1/0.2	0.1/0.4	− 0.2/0.08
**Age at diagnosis**	0.01/0.8	0.002/0.9	0.1/0.2	0.08/0.4	0.02/0.8	0.05/0.6	− 0.05/0.6	0.05/0.6	0.07/0.4
**Freq. of visits**	− 0.2/0.01*	0.2/0.03*	− 0.02/0.8	− 0.2/0.008*	0.004/0.9	0.01/0.8	− 0.06/0.5	0.04/0.7	− 0.1/0.2
**Adm. period**	0.3/0.001*	− 0.1/0.1	− 0.07/0.4	− 0.05/0.6	− 0.1/0.06	− 0.04/0.7	− 0.18/0.054	0.04/0.6	− 0.05/0.5
**Duration of treatment**	0.2/0.01*	− 0.1/0.2	− 0.1/0.2	0.08/0.3	− 0.1/0.02	− 0.01/0.9	− 0.01/0.8	− 0.1/0.2	− 0.02/0.8

*Significant at the level P<0.05

### Regression analyses

Multiple regression analyses (Table 3) showed that there was a highly significant association between all the eight-cancer-specific quality-of-life domains except the “pain” domain and the overall quality-of-life log scores in cancer pediatric patients. “Nausea” followed by “worry” and “cognitive problems” was the most effective domains on the overall quality of life score. The improvement in “nausea,” “worry,” and “cognitive problems” by 1% will improve the overall quality of life by 0.4, 0.38, and 0.34%, respectively (*P* < 0.0001). The improvements in the score of “procedure anxiety,” “treatment anxiety,” “perceived physical appearance,” and “communication” problems by 1% will be associated with a significant improvement in the overall quality-of-life score by 0.2% for each domain (*P* < 0.001).

**Table 3:** Association between the cancer-specific quality-of-life domains and the overall quality-of-life log scores

Variable	Adjusted *β*-coefficient ± standard error	*p-value*
Pain and hurt	− 0.005 ± 0.05	0.9
Nausea	0.4 ± 0.09	< 0.0001**
Procedure anxiety	0.2 ± 0.08	0.006**
Treatment anxiety	0.2 ± 0.04	< 0.0001**
Worry	0.38 ± 0.06	< 0.0001**
Cognitive problems	0.34 ± 0.06	< 0.0001**
Perceived physical appearance	0.2 ± 0.05	< 0.0001**
Communication problems	0.2 ± 0.06	< 0.0001**

^**^Correlation is significant at the 0.01 level (2-tailed)

As regards the association between each individual item of the eight-cancer-specific domains with its domain and with the overall QoL scores (TSQL), both items of “pain” domain: “joint and/or muscle pain” and “feeling lot of pain symptoms” were significant predictors for the “pain” as a subdomain (*P* < 0.0001), but not for the TSQL (*P* > 0.05). All five individual symptoms of “nausea” problems were significant predictors for the “nausea” domain (*P* < 0.0001), but only “feeling nausea during taking medication” (*P* < 0.0001) and “tendency to vomit once thinking of medication” (*P* < 0.0001) could significantly predict the overall quality-of-life score. Each improvement in the score of each one of the two problems by 1% could be accompanied by an improvement of the overall QoL score by 0.2%.

“Feeling pain because of needle sting” and “anxiety because of using needles” significantly predicted the “procedure anxiety” problem (*P* = 0.004 and 0.02, respectively). All the individual items of the “procedure anxiety” could not predict the overall QoL score (*P* > 0.05). All of the three individual symptoms of the “treatment anxiety” problem significantly predicted the score of the “treatment anxiety” problem (*P* < 0.0001). Only “anxiety because of visiting the doctor” (*P* = 0.04) and “anxiety because of going to the hospital” (< 0.0001) could significantly predict the overall quality-of-life score. It was found that each improvement in the two problems’ scores by 1% could be accompanied by an improvement in the overall score of pediatric QoL by 0.08 and 0.1%, respectively.

On the other hand, all of the three individual symptoms of the worry problem were highly significant predictors of the worry score as well as the overall score of PedsQoL. It was clear that every improvement by 1%, in the patient’s worry regarding the treatment impact, the disease relapse, or side effects of the treatment, could be accompanied by an improvement of the overall quality-of-life score by 0.4%, 0.4%, and 0.3%, respectively (*P* < 0.0001). Four out of five individual symptoms that measure the cognitive problem could predict it significantly, while the item of difficulty in deciding what to do with something that bothered him adds no significant effect on the model (*P* > 0.05).

The complaint difficulty in solving mathematical problems and difficulty in writing homework succeeded to predict the score of the overall quality of life, an improvement in the former complaints, and could improve the overall quality-of-life score by 0.2 for the first and 0.1% for the last one (*P* = 0.007 and 0.004, respectively). In the perceived physical appearance of QoL domain, all the individual symptoms could predict the domain model significantly (*P* < 0.0001). The two complaints felt that he/she is not looking well and not liking others to notice treatment effects on him/her and significantly predicted the score of the total quality of life (*P* < 0.0001). The improvement in the last complaint by 1% could improve the score of the overall quality of life by 0.3% versus 0.05% in the former one. As well, only two individual complaints of the communication problem could significantly predict the problem (*P* < 0.0001). Improving the difficulty of the patient in asking the doctor/nurse or the difficulty to explain the nature of his/her disease to others by 1% could improve the overall QoL score by 0.3% (*P* < 0.0001) and 0.1% (0.002), respectively.

## Discussion

The mean value of PedsQL™ 3.0 Cancer Module score among the study sample showed relatively low QoL (49.3 ± 12.0), when compared to values reported by Ji et al. [13] upon applying the same instrument on a group of Chinese cancer children, Sand et al. [14] on Swedish cancer children, and Hegazy et al. [15] on Saudian pediatric cancer patients (71.0, 69.0 and 73.4, respectively). Moreover, it has declined below the detected value by Fawzy et al. [12] (62.29) upon using the same tool with a comparable population in Egypt. This finding goes in agreement with the poor quality of life reported by Mounir and Abolfotouh [16] where they used the Pediatric Oncology Quality of Life Scale on a sample of school children with cancer in Alexandria (an Egyptian governorate). Yet, fortunately, our finding is still better than the previously reported value of 42.07 by Chaudhry and Siddiqui [17] in Pakistan detected by using PedsQL™ 3.0 Cancer Module.

Upon analysis of the obtained data to identify the specific items and domains of PedsQL™ 3.0 Cancer Module that mostly contributed to the poor quality of life among the study sample, some interesting insights and interpretations were obtained. Both items of the pain domain significantly contributed to the main domain but not to the total score of QoL with an indication that pediatric patients with cancer suffer from various kinds of pain that could be related to many factors and caused by various inducers yet not significantly contributing to the low state of QoL of the study sample. In accordance with our findings, the results obtained by Duran and his colleges [18], who reported no significant differences between quality of life of cancer module total and different domain scores between cancer children with and without pain, in spite the HRQoL domains may be affected by the presence of pain when assessed by the PedsQL™ generic instrument. While a previous research has demonstrated that pain is a potentially debilitating side effect associated with cancer treatment, it contributes to distress of patients and their families, awfully affects quality of life, impedes recovery, and is associated with long-term morbidity [19]. Both pain and subsequent sleep disturbance may represent contributing factors to an impaired HRQoL in children and adolescents with cancer [20, 21]. Pediatric cancer patients have suffered more pain from treatments and painful procedures than from the disease itself [22].

On the other hand, some individual items belonging to the other seven domains of the PedsQL™ 3.0 Cancer Module proved significant contributions to the total quality-of-life score (tQoL). The “worry” domain and all of its three items were significantly associated with low quality of life, and at the same time, the domain showed very low score on standing (16.6 ± 28.5). Despite the lowest score being recorded was for the “procedural anxiety” domain (8.7 ± 23.9), such domain contributed to the total score of QoL but none of its items. Such finding could give an indication of the extended effect of the worry experienced by pediatric cancer patients that was expressed in terms of their fear of disease relapse, the suspected harmful impact, and side effects of treatment on their lives both for patients under active treatment, those under remission who are taking their supportive medication, and even ALL survivors. In the same context, Tonsing and Ow [23] reported cancer-specific worry as an emerging significant predictor for overall QoL among survivors of childhood cancer.

According to our significant results, a higher frequency of hospital visits by two visits or more was associated with increased feelings of pain and treatment anxiety yet decrease in suffering from nausea and vice versa. While the longer periods of hospital admission for more than half of the recommended treatment periods were associated with reduced pain suffering on the expense of increase in feelings of worry as well as communication problems, it has also been shown that the perceived physical appearance was better among those patients who spent a treatment period for 3–6 months when compared to those who spent a treatment period less than 3 months or more than 6 months. This was in harmony with the conclusion of Hegazy et al. [15], which more pain and hurt, significant worry, nausea, and treatment anxiety were all linked to higher frequency of hospital visits, longer duration of diagnosis, longer duration of treatment, and increased therapy intensity, all of which represent a poor quality of life in pediatric cancer patients.

Thus, it is clear that treatment at hospitals could be the worst choice ever for childhood cancer patients. Home treatment has been proved to be among the most successful interventions to enhance general wellbeing and quality of life among cancer patients under treatment [24, 25].

In the present study, body mass index (BMI) was significantly inversely correlated to the QL treatment anxiety domain score; also, obese and overweight children had poor quality of life than children with normal weight. Many studies [26–28] had talked about the major impact of overweight and obesity on the quality of life of children and adolescents, from these, the study done in the National Nutrition Institute, Egypt by Khairy et al. [29], but there was lack of researches done to study the effect of weight on quality of life in cancer children.

It appears from the study results that some measures must be taken to improve QoL of children with cancer in Egypt. As concluded from previous studies, building a trusting relationship with healthcare providers could improve the quality of life among patients [30]. Other encouraging procedures to improve QoL include the following: applying a complementary Chinese medicine [31] or honey [32] in combination with the chemotherapy, engaging patients with physical activities [33], and creative arts therapy [34]. Furthermore, several researches have described that patient educational interventions help to grant patient satisfaction [35], besides increased parental knowledge about the problems and needs of their leukemic children [36]. Moreover, parental psychosocial adjustment [37] has proven a cardinal influence on the family support, thereby causing a significant quality-of-life improvement for their children [38]. Stress has been detected among many populations in Egypt including females, low-income families, and some specific jobs and professions like employees over workers, researchers, and cotton industry workforce in weaving and spinning [39–44]. Besides, the pandemic came to worsen the case and aggravate the mental health condition to target more vulnerable populations like housewives and the youth under 44 years in age. In such cases, quality of life for patients with cancer as well as their families become more prone to vast deteriorations that invite more intensive support and care [45].

As a sum, the mean value of the PedsQL™ 3.0 Cancer Module score is shown to be 49.3 ± 12.0 among 110 pediatric cancer patients that were admitted to an outpatient clinic in Egypt. The lowest mean score of quality of life was for the procedure anxiety, followed by worry. Higher frequency of hospital visits could be the cause of increased feeling of pain and treatment anxiety. On the other hand, they were useful in decreasing nausea. The longer period of hospital admission also affected QoL scores.

## Conclusion

In conclusion, cancer pediatric patients in Egypt are experiencing low quality of life concerning certain domains including anxiety procedure and worry domains. Nausea, worry, and cognitive problems affect their perception of quality of life to a great extent.

### Recommendations

HRQoL is an issue that deserves much concern for the sake of better life for pediatric cancer patients and survivors. Hence, long-term follow-up clinics for survivors of childhood cancer are highly recommended to monitor HRQoL throughout time, to help pediatric patients with cancer to deal with pain and defeat negative thoughts related to the fear of the disease, the treatment, and the threat of relapse. This could be achieved by narrowing the gap between patients and their medical staff, creating cheerful surroundings full of joy and hope, improving treatment facilities, teaching parents how to care for their kids and how to manipulate their worries, and finally by providing patient education to clarify related issue and sending calming messages through trusted specialists.

## Data Availability

The data sets used and analyzed in this study are available from the corresponding author upon reasonable request.

## Abbreviations

ALL: Acute lymphocytic leukemia
BMI: Body mass index
CCHE: Childhood Cancer Hospital of Egypt
HRQoL: Health-related quality of life
IQR: Interquartile range
M ± SD: The mean score with standard deviation
MR: The mean rank
NCI: National Cancer Institute
PedsQL™: The Pediatric Quality of Life Inventory
QoL: Quality of life
SPSS: Statistical Package for the Social Sciences
TSQL: The total score of quality of life
Vs: Versus
